# Silent Intravascular Lymphoma Initially Manifesting as a Unilateral Adrenal Incidentaloma

**DOI:** 10.1155/2012/849285

**Published:** 2012-08-13

**Authors:** Yoshiko Takahashi, Keiji Iida, Yasuhisa Hino, Takeshi Ohara, Toshifumi Kurahashi, Takashi Tashiro, Kazuo Chihara

**Affiliations:** ^1^Division of General Medicine, Hyogo Prefectural Kakogawa Medical Center, 203 Kanno, Kanno-cho, Hyogo, Kakogawa 675-8555, Japan; ^2^Division of Diabetes and Endocrinology, Hyogo Prefectural Kakogawa Medical Center, 203 Kanno, Kanno-cho, Hyogo, Kakogawa 675-8555, Japan; ^3^Department of Urology, Hyogo Prefectural Kakogawa Medical Center, 203 Kanno, Kanno-cho, Hyogo, Kakogawa 675-8555, Japan; ^4^Department of Pathology, Hyogo Prefectural Kakogawa Medical Center, 203 Kanno, Kanno-cho, Hyogo, Kakogawa 675-8555, Japan

## Abstract

Intravascular large B-cell lymphoma (IVLBCL) is a rare subtype of malignant lymphoma. Although the involvement of adrenal glands in IVLBCL is often observed, primary adrenal IVLBCL is rare. Most reported cases of adrenal IVLBCL showed bilateral lesions resulting in rapidly progressive adrenal failure and poor prognosis. Here, we report a case of slowly progressive primary adrenal IVLBCL manifesting initially with unilateral adrenal incidentaloma. This case is a silent IVLBCL and shows that the enlargement of both adrenal glands can be followed.

## 1. Introduction

Intravascular large B-cell lymphoma (IVLBCL) is a rare type of malignant lymphoma characterized by proliferation of malignant lymphoid cells within the lumens of small-to medium-sized blood vessels [1, 2]. Although the brain, skin, and lung are the most frequently involved organs in IVLBCL, other organs, including the adrenal glands, have been found to be involved in autopsy cases [3, 4]. Most of the reported cases of adrenal IVLBCL showed development of bilateral lesions resulting in rapidly progressive adrenal failure. Such cases had poor prognosis and were frequently undiagnosed until the time of autopsy [2, 5]. We report here a rare case of silent primary adrenal IVLBCL initially manifesting as a silent adrenal incidentaloma. We were able to follow the morphological change of both adrenal glands during the one year follow-up period.

## 2. Case Presentation

**Figure 1:**
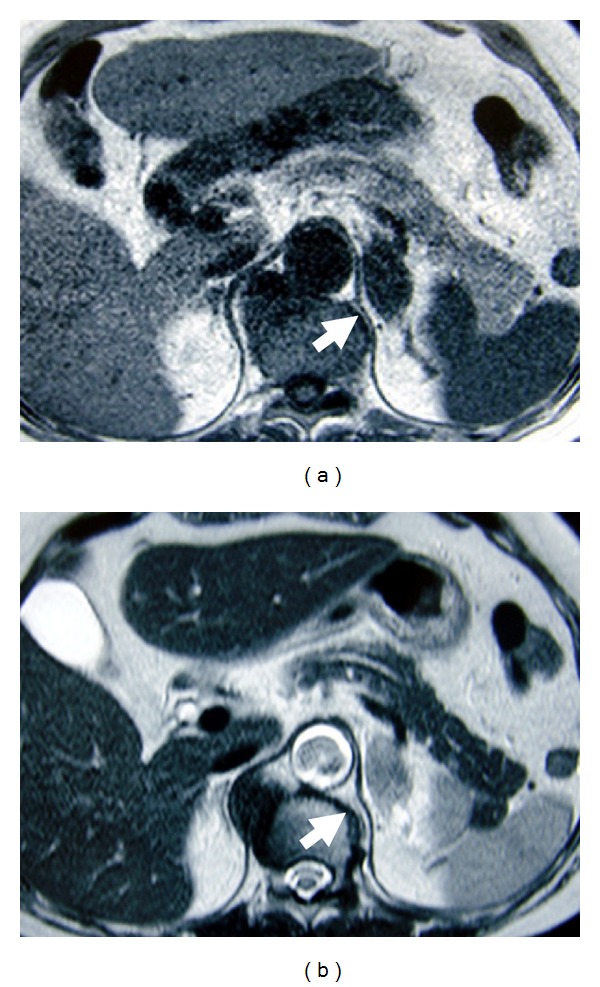
T1-weighted (a) and T2-weighted (b) MRIs of the enlarged left adrenal gland (arrowhead) demonstrating a low- and isointensity signal, respectively. The right adrenal gland was normal in size and shape.

**Figure 2:**
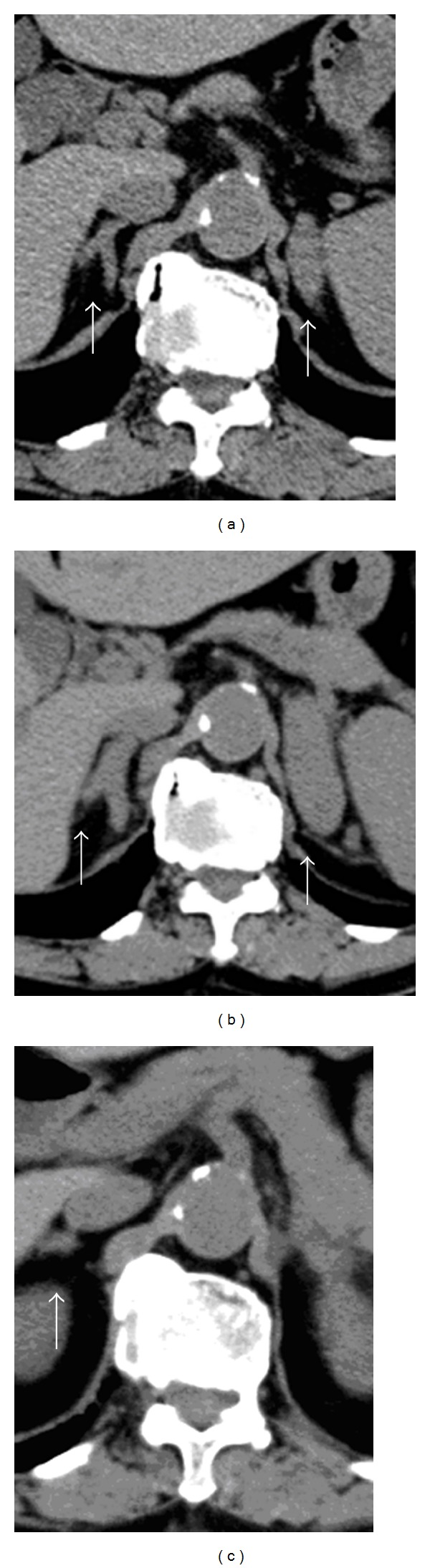
CT image (a) on the first visit; (b) 1 year after the first visit revealing enlarged adrenal glands; and (c) after R-CHOP therapy showing shrinkage of the right adrenal gland.

**Figure 3:**
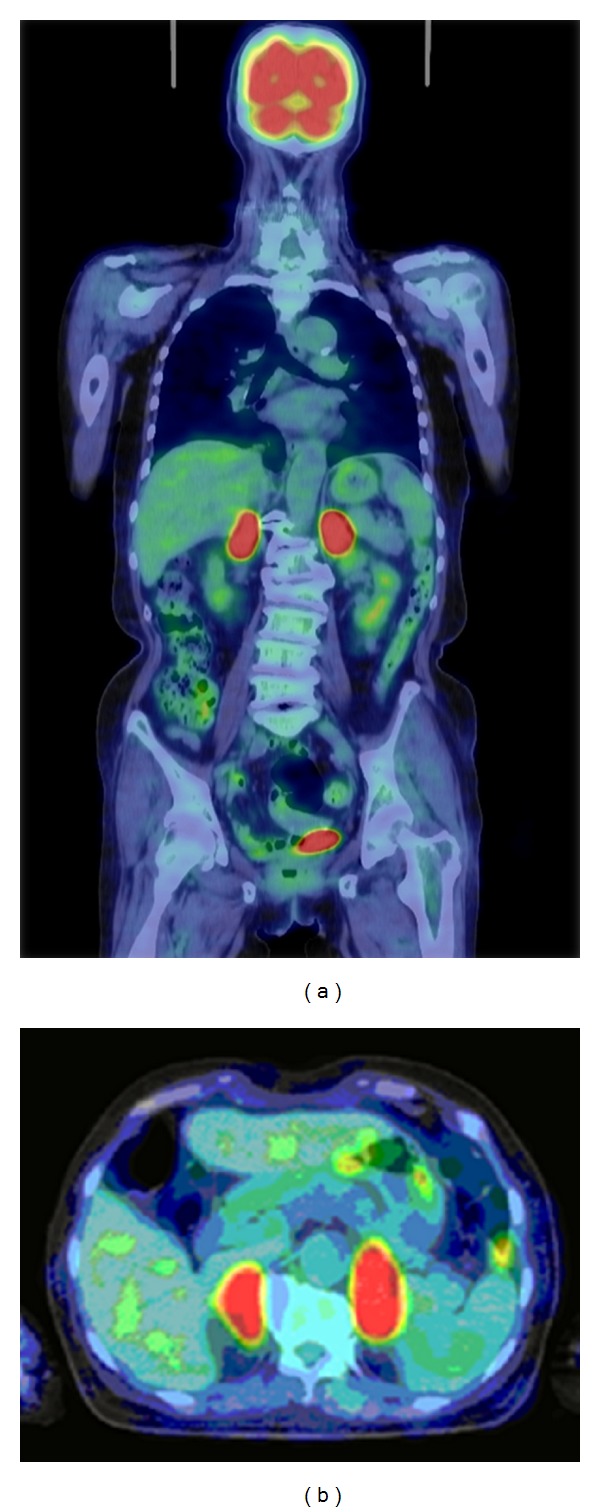
FDG-PET image showing an exclusive strong uptake in both adrenal glands: (a) coronal section; (b) horizontal section.

**Figure 4:**
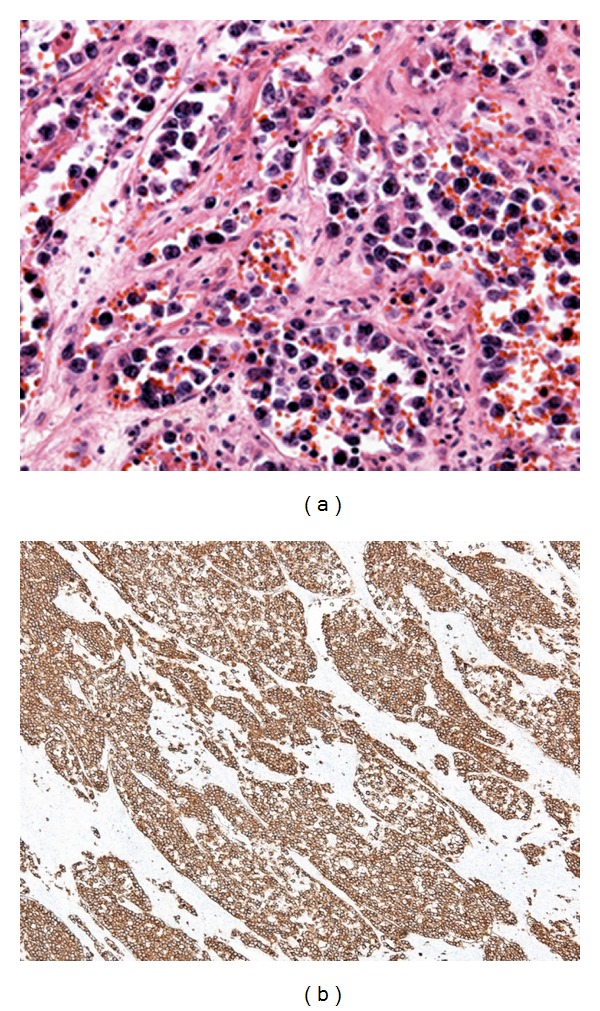
Histological examination of the specimen obtained by adrenalectomy. (a) Atypical lymphocytes with large nuclei were mainly infiltrating into the lumina of small vessels. (b) Infiltrating cells were positive for CD20.

A 75-year-old woman visited our hospital presenting with temporary chest pain. She had hypertension and type 2 diabetes mellitus (T2DM) treated with calcium antagonist and metformin, respectively, by her family physician. Physical examination revealed that she had neither lymphadenopathy nor hepatosplenomegaly. Electrocardiogram revealed no signs of acute coronary syndrome (data not shown). Slight elevation of serum lactate dehydrogenase level as well as anemia was observed. Enlargement of the left adrenal gland was identified incidentally by abdominal computed tomography (CT), whereas her right adrenal gland was normal in size and shape. Her left adrenal gland was elliptically shaped, with the longest diameter being 35 mm. As shown in Figure 1, the T1- and T2- weighted views in the magnetic resonance image (MRI) of her enlarged left adrenal gland showed a low- and isointensity signal, respectively. Because she has hypertension and T2DM, we first suspected a functioning adrenal tumor such as Cushing's syndrome, primary aldosteronism, or pheochromocytoma. However, endocrinological examinations revealed that her adrenal function was normal. Her serum ACTH and cortisol levels at that time were 34.3 pg/mL and 8.7 *μ*g/dL, respectively. Administration of 1 mg dexamethasone at midnight suppressed her serum cortisol level up to 2.8 *μ*g/dL in the next morning, indicating that there was no autonomous cortisol production from the adrenal tumor. Primary aldosteronism was unlikely because her serum renin activity (0.5 ng/mL/hr) and aldosterone levels (78.5 pg/mL) were both within normal limits. Her serum adrenaline, noradrenaline, and dopamin levels were 30 pg/mL, 306 pg/mL, and 11 pg/mL, respectively, indicating that adrenal tumor was not typical with pheochromocytoma. Six months later, the size of her adrenal glands was unchanged. However, the CT scan at the next 6 months revealed obvious enlargement of both adrenal glands (Figure 2). Her serum ACTH and cortisol levels at that time were 96.6 pg/mL and 10.2 *μ*g/dL, respectively, indicating that her adrenal function was still normal. Her serum level of soluble interleukin-2 receptor (sIL2R) was 5,930 U/mL, and the exclusive strong uptake in both adrenal glands in F-18 fluorodeoxy-glucose positron emission tomography (FDG-PET) (Figure 3) was observed. We suspected adrenal tuberculosis infection, adrenal metastasis from unknown primary cancer, or malignant lymphoma. To make a definite diagnosis, laparoscopic left adrenalectomy was performed. Histological examination demonstrated diffuse infiltration of atypical lymphocytes, with large nuclei not only replacing normal adrenal structure but also mainly proliferating into the lumina of small vessels. Immunohistochemical staining demonstrated that most tumor cells were positive for B- cell markers such as CD20 (Figure 4), bcl-6, and multiple myeloma oncogene 1 (MUM1), whereas those same tumor cells were negative for CD3, CD5, CD10, or bcl-2 (data not shown). Taking the findings together, a final diagnosis of IVLBCL was concluded. Bone marrow examination revealed no lymphoma cells but a hemophagocytosis (data not shown). 

As treatment, CHOP therapy at a reduced dose was started due to her advanced age. We used 600 mg/m^2^ of cyclophosphamide (80% of standard dose), 40 mg/m^2^ of adriamycin (80% of standard dose), 1.12 mg/m^2^ of vincristine (80% of standard dose), and 100 mg/body of prednisolone (standard full dose) for 5 days, combined with 375 mg/m^2^ of rituximab (standard full dose) on the first day (R-CHOP). After the 8th cycle of R-CHOP therapy, her right adrenal gland decreased in size (Figure 2) and her serum sIL2R levels were decreased from 5,930 U/mL to 734 U/mL. One year after R-CHOP therapy, FDG-PET scan revealed disappearance of radioactivity in her right adrenal gland (data not shown), indicating that chemotherapy led her to the condition of complete remission.

## 3. Discussion

In this paper, we describe a rare case of primary adrenal IVLBCL that initially showed silent unilateral adrenal swelling. That we could detect IVLBCL at the early phase and observe the process of morphological change of both adrenal glands in a patient with primary adrenal IVLBCL is a noteworthy finding. 

Several reports of IVLBCL cases have been published since the first case was reported in 1959 [6]. The reported clinical features of IVLBCL are modestly different between Western and Asian countries [7]. Caucasian patients display a relatively high frequency of central nervous system and skin involvement, while in Asian patients, bone marrow involvement, fever, hepatosplenomegaly, and thrombocytopenia are more common (referred to as Asian variant IVLBCL) [7]. Our case might be partly consistent with the Asian variant IVLBCL, as tumor cells were exclusively found in the adrenal glands as suggested by FDG-PET as well as by the absence of skin lesions or neurological abnormalities. 

Adrenal involvement is thought to be relatively common in patients with IVLBCL [1, 5]. This is in a sharp contrast with the finding that adrenal involvement in disseminated non-Hodgkin's lymphoma is rare and occurs in only 4% of cases by CT scan [8]. Although the mechanism of the preferential involvement of lymphoma cells to the adrenal glands in IVLBCL remains unknown, its association with a lack of homing receptors and adhesion molecules, including CD29 (*β*1 integrin) and CD54 (intercellular adhesion molecule 1), has been hypothesized [9, 10], and this might be one of the distinct characteristics of IVLBCL.

Several previously reported cases showed bilateral adrenal enlargement with adrenal insufficiency as the initial manifestation of IVLBCL [11]. Furthermore, approximately 50% of the reported cases with primary adrenal IVLBCL were not recognized clinically and the correct diagnosis was made only at autopsy, owing to the rapidly deteriorating clinical course [11, 12]. Contrary to the previously reported cases, the present case showed a silent clinical characteristic, as well as unilateral adrenal incidentaloma without fever, general fatigue, or adrenal insufficiency. Abdominal CT scan at a periodic checkup revealed asymptomatic bilateral enlargement of adrenal glands, while her adrenal function remained normal, suggesting a relatively slow progress of the disease.

In treatment of IVLBCL, standard CHOP regimen is practically used in many cases. Furthermore, the additional administration of rituximab, a recombinant monoclonal antibody to CD20, to the standard CHOP therapy is reported to ameliorate the outcomes of IVLBCL treatment in Japan [13] as well as in Europe [14]. We treated our patient with 8 cycles of a reduced dose of R-CHOP regimen, and her enlarged right adrenal gland shrank to normal size (Figure 2). She maintained a good condition of complete remission one year after the chemotherapy, in agreement with previous reports [13, 14].

In conclusion, we presented a rare case of clinically silent primary adrenal IVLBCL, wherein we were able to observe the process of enlargement of both adrenal glands.
